# Advances in Sucker Control for Sustainable European Hazelnut (*Corylus avellana* L.) Cultivation

**DOI:** 10.3390/plants11243416

**Published:** 2022-12-07

**Authors:** Alberto Pacchiarelli, Cristian Silvestri, Valerio Cristofori

**Affiliations:** Dipartimento di Scienze Agrarie e Forestali (DAFNE), Università della Tuscia, Via San Camillo De Lellis s.n.c., 01100 Viterbo, Italy

**Keywords:** *Corylus avellana* L., suckers’ management, herbicides, non-suckering rootstocks, grafted plants

## Abstract

European hazelnut (*Corylus avellana* L.) is a shrub native to temperate zones of the northern hemisphere, and it is the most important species among the *Corylus* genus, mainly due to its high kernel demand from the confectionery industry. Its spontaneous habitus is characterized by a bushy shape, formed by numerous lignified stems generated by seasonal emission of suckers, which develop from adventitious buds inserted in the collar of stems, or more generally from the stump. Despite the agronomic role of suckers, which are often used to replace diseased, old, or poorly oriented branches, they compete with the plant for water and nutrient uptake, negatively influencing its growth and yield. In addition to promoting mechanical applications during the hazelnut orchard management, sucker removal is a required agronomic operation that must be carried out yearly during the growing season, making this practice expensive and time consuming, especially when performed manually. To date, there are several techniques for hazelnut sucker management, and their application depends on several factors, such as the size of the farm, model of cultivation (conventional or organic), soil orography, and plant training system. This review discusses the most widespread methods applied for sucker control, including manual, mechanical, physical, and chemical control (flame and steam), use of non-suckering rootstocks, disbudding, mulching, nitrogen solution applications, and new automatized control techniques recently proposed at the experimental level, analyzing their advantages and disadvantages.

## 1. Introduction

European hazelnut (*Corylus avellana* L.) is one of the most important species among the nut trees [1], and the steadily increasing demand for hazelnuts by the confectionery industry at global level has led to continuous expansion of hazelnut cultivated areas in recent years, both in historically suitable areas (Turkey, Italy, Spain, Georgia, and the USA) and in new countries such as Australia, South Africa, and Chile [2,3]. To date, hazelnut cultivation covers over 700,000 harvested hectares worldwide, and the yearly average in-shell nut production is mainly concentrated in the following countries: Turkey (665,000 t), Italy (140,560 t), the USA (64,410 t), Azerbaijan (49,465 t), Chile (33,939 t), Georgia (32,700 t), and China (24,263 t) [4].

The continuous formation of new herbaceous suckers at the base of the collar of woody stems or plant stumps during the growing season is the consequence of its typical bushy development [5]. To date, the bush training system is still widespread in hazelnut cultivation, especially in the Mediterranean basin and in Turkey [2].

European hazelnut is a species with high suckering emission aptitude, and the number of suckers emitted varies among cultivars [6], such that the main hazelnut varieties have been classified according to their sucker’s emission aptitude [7,8]. However, suckering aptitude is not exclusively determined by genotype; it can also be influenced by additional factors, such as training system, planting layout, seasonal weather conditions, and biennial bearing aptitude of the species.

In the past, the sucker emission was considered of economic value as it was functional to the gradual renewal of the orchard and it allowed on-farm supply self-rooted plants to be used to plant new hazelnut orchards.

Furthermore, this idea was consolidated, as hazelnut plants trained as multi-stemmed bush play a preventive role against soil erosion phenomena [1].

Despite these advantages, in the new-generation hazelnut orchards with high levels of mechanization, the presence of suckers is detrimental for several reasons, including competing with the plant canopy for nutrients and water [9], causing a reduction in plant growth, extending the juvenile phase, and reducing nut yield [10]. In addition, suckers can decrease the ventilation capacity of the plant, promoting environmental conditions conducive to disease development [11], and they may hinder the orchard management (chopping, fruit picking, etc.).

For these reasons, from the second year after planting, sucker removal is an essential practice. During the juvenile stage of the plants, suckers are removed manually, taking special care to remove only those in excess and with poor growth and orientation, thus promoting the development of the future bush, leaving 4–5 vigorous and well-oriented suckers to develop.

From the fourth year, control strategies can be carried out manually or mechanically, although the time-consuming nature and high cost of this operation [12,13], increasingly accompanied by a lack of skilled labor, makes chemical sucker control using authorized herbicides the most widespread practice [1]. 

Chemical suckers’ management can be carried out during their herbaceous stage, and usually at least a couple of interventions during the growing season are needed due to the sucker regrowth, or later in the season when suckers are lignified mainly using physical techniques [11].

The aim of this work is to review the techniques used to date in sucker control, as adopted by major producing countries globally, further discussing the main advantages and disadvantages of each individual technique.

## 2. Manual Control

In traditional Italian and Turkish hazelnut districts, manual sucker control is still largely applied, even if this cultural operation requires high labor force influencing up to one-fifth of the annual orchard management costs [9,13]. Manual sucker removal performed with proper tools such as shears or small hoes may be considered highly suitable for small-scale and organic farms.

This practice is usually carried out in a single summer operation, as this is a time when seasonal suckers have slowed down their growth rate and tend to lignify. 

The main advantages related to manual sucker control include its status as an ecological method and promotion of the gradual rejuvenation of the plant stump in the orchards grown on a multi-stemmed bush, a feature particularly appreciated by the grower [1]. 

Contrariwise, the main disadvantages associated with this technique, in addition to those already mentioned and the opening of wounds in the collar area that may favor the penetration of pathogens, are also related to manual removal of suckers being ergonomically dangerous and tiring work, since the repetitive pruning movements and prolonged bending could cause occupational injuries [14].

## 3. Mechanical Control

Managing suckers in organic hazelnut orchards farming, in addition to manual control, can be achieved using mechanical approaches.

This practice can be applied both to plants trained as single-trunk and to shrubs, taking care during removal operations to avoid damages on the main branches.

This type of control is mainly carried out using appropriate shoulder-mounted brush cutters equipped with cutting nylon line or metal disk, a flail mower with a lateral rotary cutter on a swing-arm linkage or using an orchard-vineyard de-suckering machine equipped with a horizontally rotating drum with a whipping brush of nylon strings [15]. 

Mechanical control is a quick practice that can be replicated with multiple seasonal interventions on suckers at the herbaceous stage. 

When applied on plants trained as multi-stemmed bushes, it does not allow all suckers to be removed, especially those inserted in the inner portion of the bush [15].

The application of mechanical sucker control determines a significative reduction in labor (up to 55%) and costs (up to about 20%) compared to manual control [13]. Moreover, mechanical control can be used as a complement to chemical control if some suckers have resisted the phytotoxic activity of the suckering herbicide.

## 4. Physical Control

Along with manual and mechanical control, physical management is another agronomic practice useful for suckers’ removal, especially in organic farms. Water steam and fire (open flame) administered by means of proper machines attached to the tractor are the two thermal procedures used for physical control of suckers. These techniques have been extensively studied in Italy by [11,15], with the aim of comparing both treatments and establishing their effectiveness, economic feasibility, and potential damage to the main branches or plant stumps.

The application of these methods consists of a short-lasting jet of intense heat sprayed directly onto the herbaceous suckers, causing them to wither and die a few days after treatment [16].

This control method can be carried out on suckers both in herbaceous and lignified stage. If applied early (during the herbaceous stage), at least two to three interventions are necessary during the growing season due to the fast regrowth of new suckers after treatment. On the other hand, if the treatment is carried out on lignified suckers, manual removal of wilted suckers will be necessary. Further, this practice is considered very attractive, as it is environmentally friendly.

Results obtained by [11] showed that both methods have enough efficiency in suckering without damaging the plants. 

Despite the effectiveness of both practices, the water-vapor method is more complicated due to a very low working speed, the high amount of water and fuel needed, and very expensive equipment.

In contrast, flaming is easier, since both equipment cost and fuel consumption per year and hectare are quite low. Furthermore, when using flaming it is recommended to carry out the operation in the early morning and early spring, when the grass is still green, to avoid potential fires [15]. 

In conclusion, these techniques, if applied in automatic mode using tractors, can only be carried out on flat or slightly sloped surfaces, while if applied by operators, high specialization and experience is requested. Lastly, applying physical control of suckers does not allow plant rejuvenation, as all treated suckers will have the same course (wilting and then death).

## 5. Chemical Control

**Figure 1:**
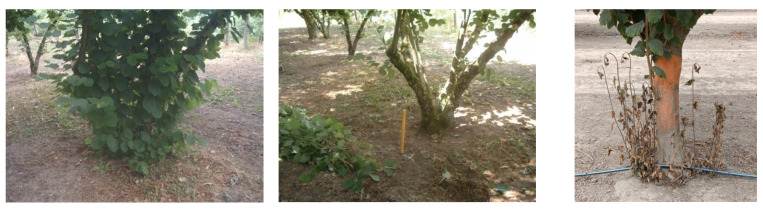
Comparison of manual and chemical hazelnut suckering (on the **left**: sucker canopy in multi-stemmed bush before manual suckering; in the **middle**: same multi-stemmed bush after manual suckering carried out in early summer; to the **right**: hazelnut suckers in wilted stage after chemical treatment).

The use of herbicides for suckering (Figure 1) is the most widely used control technique in conventional agriculture due to its main advantages, such as quick implementation and lower cost compared with other sucker control methods [1]. 

Despite these benefits, chemical control of suckers highlights problems associated with the environmental pollution, induces depression of soil microbial activity, and may lead to phytotoxicity on the crop [17]. In addition, the use of herbicides for both weed control and suckering promotes soil erosion, especially in areas characterized by slopes [11].

Generally, the number of seasonal herbicide applications for sucker control varies from two to four, depending on the climatic condition, the age of the orchard, the plant training system, the sucker emission aptitude of the cultivars [6], and the persistence of suckering herbicide (Table 1). Treatments must be carried out quickly when the suckers are in the herbaceous phase and reach about 15–20 cm high, as their development is not uniform (they show high variability in height).

If suckers are allowed to grow during the season until they lignify, many more seasonal interventions will be required to control them, often assisted by other control methods [18].

Further, [19] points out that the effectiveness of herbicides in sucker control is not only related to their development and to the number of herbicide interventions, but also to the size of spray droplets. In support, several studies shows that a reduction in droplet diameter is positively correlated with an increase in herbicide efficacy [20].

The first trials on the application of active ingredients for sucker control were conducted in Italy and Oregon (USA) in 1960 [1], although the first attempts to eliminate suckers by chemical control were carried out directly by farmers in Oregon in the first half of the 1950s [21,22].

In the early experimental years, many researchers conducted trials in which the degree of phytotoxicity of active ingredients used for sucker control was assessed, including aminotriazole, bromacil, chlorthiamid, dichlobenil, paraquat, dinoseb, diquat, cypromid, cacodilic acid, 2,4,5-trichlorophenoxyacetic acid (2,4,5-T), dicamba, 2,4-dichlorophenoxyacetic acid (2,4-D), and picloram [23,24].

Later, some studies carried out in Italy, and revised by [17,25], focused on the evaluation of additional herbicides as 1-naphtaleneacetic acid (NAA) esters and glufosinate-ammonium.

Given their high environmental impact, some active ingredients listed above are no longer authorized, such as Paraquat (N,N′-dimethyl-4,4′-bipyridinium dichloride), which was banned in Europe in January 2015 due to its harmful effects on avifauna and insects. Nevertheless, its use is not forbidden in the United States, where it is still used for suckering. Similar to Paraquat, glufosinate-ammonium was also authorized as herbicide in the EU until 2018, after which it was banned due to its assumed reprotoxic effects.

Despite the high efficacy of 2,4-D in sucker removal [26], during the summer, when treatments are often carried out under conditions of high temperatures and low air humidity, the risk of active ingredient volatilization increases significantly with a high probability of damaging the plants [27,28].

This issue could be overcome thanks to the use of NAA, which is a non-volatile compound [29]. NAA is a synthetic plant growth regulator within the auxin family that acts as an herbicide by promoting the production of abscisic acid and hydrogen peroxide, causing growth inhibition or wilting, tissue necrosis, and, consequently, death of the affected plant tissue [30].

Recently, [17,25] demonstrated the effectiveness of different derivatives of NAA in suckering without highlighting plant phytotoxicity issues or yield decline. Furthermore, other updates supporting their reports were obtained by [28], confirming that NAA performs satisfying suckering without causing direct damage to the plant. In addition, application of herbicides in solution with NAA could improve suckering when compared with treatments made individually with NAA or herbicides, with the effect of reducing the number of treatments to be performed.

## 6. Non-Suckering Rootstocks

A single-trunk training system is increasingly popular in both major and new hazelnut-producing countries to facilitate the management of commercial hazelnut orchards. It also allows planting at higher densities. 

As a consequence of adopting single-trunk systems, the plants have an increased sucker emission aptitude, demonstrating an increased commitment to their control.

The use of non-suckering rootstocks may be a solution to overcome several issues associated with higher plant sucker emission when hazelnut is trained as a single trunk.

To date, the most used non-suckering rootstocks are seedlings of open-pollinated *C. colurna* trees obtained by seed germination directly harvested in the forests, as is routine in Serbia, and some clonal hybrid selections released in 1990s by the hazelnut breeding program carried out in the Oregon State University [31].

First attempts to use *C. colurna* seedlings as non-suckering rootstocks in European hazelnut were carried out at Oregon State University (Corvallis, OR, USA), over the period 1940–1970, during which the main advantage, besides there being no sucker emission, was the deeper root systems of the *C. colurna* seedlings in comparison to that of self-rooted *C. avellana* plants. At the same time, some disadvantages also emerged, such as the long germination time requested by the seedlings (until two years), the slow initial growth that adversely affected the plants’ suitability for grafting (two additional years after germination to get the right grafting size), and their poor vegetative propagation aptitude [32]. Due to these emerged issues during the trials, at that time, *C. colurna* seedlings were considered unsuitable as rootstocks for European hazelnut grown in the main producing areas of the United States [33].

A few years later, the same attempt to use *C. colurna* seedlings as non-suckering rootstocks started in Serbia through the field evaluation of some seedling populations obtained from field germination of *C. colurna* seeds harvested from wild trees [34,35]. 

From these assessments, two genotypes were selected for use as rootstocks, namely NS A2 and NS B4, which showed high vigor and more suitability to low temperatures and drought conditions [36,37,38,39].

In addition, at Oregon State University in 1968, coinciding with the start of the hazelnut breeding program, hybrid seedlings obtained from emasculated mature trees of *C. colurna* open-pollinated with mixed pollen of *C. avellana* were tested. Two interspecific hybrids exhibiting intermediate traits between *C. colurna* and *C. avellana* and very low sucker emission were selected and released as clonal rootstocks, named Newberg (USOR 7–71) and Dundee (USOR 15–71), respectively [40]. Unfortunately, these selections were highly susceptible to Eastern Filbert Blight, caused by *Anisogramma anomala* (Peck) E. Müll. [41], which is considered a fungal disease in the hazelnut districts of United States [33], and thus this plant material did not see wide use as non-suckering rootstock in the U.S. environment.

More recently, further investigations using these hybrids and other plant materials were carried out at IRTA-Mas Bove Research Centre (Tarragona, Spain), where an extensive field evaluation conducted over the period 2000–2015 assessed agronomic and eco-physiological behavior of one clone of the main Spanish cultivar (Negret-N9) grafted onto Dundee and Newberg rootstocks. The outputs of the trials highlighted as the grafted plants, especially those grafted onto Dundee, showed increased vigor and yield, very low sucker emission, and higher tolerance to iron chlorosis in comparison to self-rooted plants of Negret-N9 [5,42,43]. Thanks to these results, in recent years, Dundee has been used in Spain, among other countries, to achieve new grafted commercial hazelnut plantations to be grown as a single trunk.

Currently, the hazelnut rootstock propagation is attracting much attention from the nursery industry. In addition, to meet the huge demand for plant material, the propagation of hazelnut rootstocks is also being successfully carried out through micropropagation, as reported by [31].

## 7. Nitrogen Solution Applications for Suckering

Nitrogen is an essential macronutrient for hazelnut, both during the early growth stages in young plants and in mature ones. Calibrating the correct amount of nitrogen, supplied to the plant through fertilization, is extremely important to ensure proper plant development and yielding, whereas excessive nitrogen administration could have adverse plant effect, determining vegeto-reproductive imbalances. 

Despite this, recently, [13] evaluated the effect of two different nitrogen fertilizers: ammonium sulfate and calcium ammonium nitrate (21% and 26% in nitrogen content, respectively), applied in different solutions (0, 10, 15, and 20%, respectively), to determine the potential phytotoxic effect on hazelnut suckers. 

Among the different treatments carried out, the 10% solution of ammonium sulfate showed the highest efficiency in sucker control, which was comparable to that achieved using authorized herbicides. In addition, no detrimental effects of nitrogen solutions on yield, nut quality, shoot development and soil characteristics were recorded. Further, the application of nitrogen solution seems to have a positive influence on plant yield [13]. However, it remains to be verified whether nitrogen sprayed very close to the stump or plant collar, after having expressed the suckering effect, does not contribute to feeding new suckering regrowth.

## 8. Mulching

The first field trials testing the polyethylene films to prevent or otherwise reduce hazelnut sucker growth both in plants trained as a single trunk and a multi-stemmed bush were established in Italy in the 1970s [44]. 

This agronomic practice proved to be suitable for containing suckers, causing their initial altered growth, and interrupting their development at an average height of 5–10 cm, eventually leading to sucker death. 

Promising results were obtained for both training systems, although for multi-stemmed shrub, the inability to make a cover in the inner part of the stump favors a high development of suckers in this stump portion. Mulching was also able to preserve soil moisture content and keep the covered area around the plant free of weeds.

More recently, further trials on the application of plastic films around the plant and among the rows were tested in Gorbea (southern Chile) with the aim of replacing traditional weed and sucker management through mechanical or chemical methods.

Application of black polypropylene film to cover the surface around the single-trunk hazelnut plants allowed us to significantly reduce the weed and sucker growth and helped prevent soil erosion [45].

However, this sucker control strategy has not been widespread due to the limited durability of plastic films, which had to be replaced frequently, and the simultaneous advent of first-generation harvesting machines, which damaged the plastic ground covers during harvesting operations.

Recently, other mulching solutions using hazelnut shells have been explored, as these by-products used to cover the stump and the row (in this case to control weeds) to promote the soil moisture conservation, as well as during their decomposition, do not cause changes in soil pH. Covering the soil with chopped shells proved to be very useful both for soil health, by preventing weeds, and as a source of micronutrients during their decomposition, but was less effective when tested as a strategy to control suckers [46,47].

## 9. Disbudding

Removal of meristematic tissue at the base of the rooted layer in young nursery-grown plants, with the aim of evaluating a new method of sucker control, was tested by [48]. This technique, which can be promoted in organic farming, is framed as a physical sucker control method. Among the three treatments applied during the study (1—leaf removal, 2—only bud removal, and 3—meristem removal), the plants in which meristematic tissue removal was performed showed the best efficiency in sucker control. After two years of planting, the plants did not still have sucker emission. Application of this technique at nursery level before planting seems to be a promising method to control suckering, at least during the first few years after planting.

## 10. Precision Agriculture Application for Sucker Management in Large Hazelnut Orchards

Chemical hazelnut suckering is extensively performed using tractor-activated sprayers equipped with lances or bars for weed control.

Often, when using sprayers, a constant amount of suckering herbicide per plant is applied, neglecting the real need of each plant based on its quantity of suckers, which in the same orchard can be very heterogeneous, making this method even more environmentally impactful. 

A new more environmentally friendly approach to correctly distribute herbicide calibrated to the actual number of suckers per plant was recently proposed and developed as part of a European project (Project PANTHEON, funded by the European Community Horizon 2020 program under grant agreement 774571) aimed at applying precision farming techniques in large hazelnut orchards [49]. The new approach is set to release an automatized solution for suckering using robotic platforms as promising direction towards automatization in agriculture, since they provide flexible in-field actuation capabilities and potentially allow to perform several agricultural operations such as pruning [50], weeding [51] and harvesting [52].

To calibrate the amount of herbicide to be spread in each plant according to its specific needs, and to allow automatic sucker management, a mathematical model (end-to-end algorithm) to detect and quantify the real volume of suckers was developed. 

Suckering control solution is composed of two steps. In the first step, the characterization and estimation of sucker canopy dimensions of every plant is carried out. In the second step, tailor-made treatments are computed and applied to allow the application of different rates of herbicide to each tree. This innovative solution may consistently reduce herbicide volumes and enhance plant health.

The autonomous sucker management architecture consists of the following steps:

1. Design of a real-time data-driven detection system based on RGB image and respective depth map;

2. Development of an integrated segmentation and reconstruction system which generates 3D meshes of the suckers and their canopy area;

3. Design of an herbicide estimation method which exploits the estimated canopy area. Additionally, the spraying time was calculated and the movement of the sprayer (the sprayer lance equipped with proper nozzle was positioned in the desired configuration to aim the suckers, and the atomizer was activated for the estimated amount of time in order to release the herbicide);

4. Design of an autonomous spraying and aiming system for the application of the needed herbicide to the plants;

5. In-field collection of an RGB-D dataset for automatic sucker detection;

6. Full in-field validation of the approach in a hazelnut orchard using a SHERPA-HL ground-based robotic platform equipped with a RGB-D camera.

This automated detection system and autonomous sucker management approach seems very promising for application in large farms able to implement Precision Agriculture approaches in hazelnut orchard management.

## 11. Conclusions

The evaluation of different hazelnut suckers control methods aims to define the most environmentally and economically sustainable methodology to date. 

Suckering aptitude of the European hazelnut has been considered for long time as a resource for growers, given the use of selected suckers in the plant rejuvenation and in replacing dead, diseased, or poorly oriented branches and for the on-farm collection of self-rooted suckers to be used for new plantings. Despite this, modern and mechanized hazelnut cultivation requires their annual removal, as early as the second year after planting. 

Among the most sustainable control methods applicable on organic farms, the one without environmental impact is manual or mechanical control, where the exclusive use of small field tools (shears or small hoes) or brush cutters allows suckers to be managed without the use of synthetic substances. This practice still widespread in Turkey and partially in Italy and Spain. It is time consuming and expensive (it requires about 20 work hours per hectare in multi-stemmed bush orchards at a planting density of 400–500 plants/ha).

Other suckering methods, such as mulching and physical control, have found acceptance, especially in organic farms and in orchards trained as a single trunk, thanks to the absence of environmental impact. However, their efficiency is greatly reduced when applied in multi-stemmed bushes, where the suckering in the inner portion of stumps is very limited. Moreover, physical control with open flame usage could damage the basal portion of the main branches, requiring high worker expertise in carrying these field applications [1,11].

Despite the demonstrated efficiency of the disbudding procedure, the main drawback associated with this method derives from the risk of leaving viable buds in the portion of sucker subjected to the treatment, thus making further control necessary to maintain high operation efficiency [48].

Advances associated with chemical suckering, such as speed of execution and cost-effectiveness of treatments, are counterbalanced by several drawbacks, including progressive reduction over the years in the number of active ingredients available (especially in Europe) and in their related effectiveness. Another negative aspect associated with the use of herbicides is undoubtedly the high environmental impact caused by their application. For these reasons, the search for alternative or complementary solutions, which can increase the sustainability of this practice, is necessary [31]. In this regard, the possibility of interventions with salt-effect products such as spraying high concentrations of nitrogen solutions to replace herbicides is supported [13]. 

However, replacing them with additional chemically derived products, such as fertilizers, does not seem to be the most suitable alternative to make this cropping operation sustainable. Indeed, the use of excessive amounts of nitrogen solutions could lead to increased nitrate content in groundwater, brought about by leaching and percolation phenomena, resulting in issues related to groundwater healthiness [53].

Thus, from the comparison of the different techniques applied in hazelnut suckering, the use of clonal rootstocks and the application of calibrated amount of herbicides, as proposed by recent precision agriculture applications in large hazelnut orchards, seem to be the most promising methods driving sustainable intensification of the hazelnut orchard.

## Figures and Tables

**Table 1:** Suckering herbicides authorized in Europe and USA (AI = active ingredient).

AI	Trade Name	Authorized AI	
		Europe	USA
Carfentrazone ethyl	Affinity plus; Spotlight plus	Yes	No
Pyraflufenethyl	Evolution; Piramax EC	Yes	No
2,4-D	Saber	No	Yes
Carfentrazone	Aim EC	No	Yes
Glufosinate	Rely 280	No	Yes
Paraquat	Gramoxone SL	No	Yes
Pelargonic acid	Scythe	No	Yes
Pyraflufen	Venue	No	Yes
